# Relative clause comprehension in Cantonese-speaking children with and without developmental language disorder

**DOI:** 10.1371/journal.pone.0288021

**Published:** 2023-11-07

**Authors:** Jane Lai, Angel Chan, Evan Kidd

**Affiliations:** 1 Department of Chinese and Bilingual Studies, The Hong Kong Polytechnic University, Hong Kong, China; 2 Research Centre for Language, Cognition, and Neuroscience, The Hong Kong Polytechnic University, Hong Kong, China; 3 Peking University Research Centre on Chinese Linguistics, The Hong Kong Polytechnic University, Hong Kong, China; 4 Max Planck Institute for Psycholinguistics, Nijmegen, The Netherlands; 5 The Australian National University, Canberra, Australia; 6 ARC Centre of Excellence for the Dynamics of Language, Canberra, Australia; COMSATS University Islamabad, PAKISTAN

## Abstract

Developmental Language Disorder (DLD), present in 2 out of every 30 children, affects primarily oral language abilities and development in the absence of associated biomedical conditions. We report the first experimental study that examines relative clause (RC) comprehension accuracy and processing (via looking preference) in Cantonese-speaking children with and without DLD, testing the predictions from competing domain-specific versus domain-general theoretical accounts. We compared children with DLD (N = 22) with their age-matched typically-developing (TD) children (AM-TD, N = 23) aged 6;6–9;7 and language-matched (and younger) TD children (YTD, N = 21) aged 4;7–7;6, using a referent selection task. Within-subject factors were: RC type (subject-RCs (SRCs) versus object-RCs (ORCs); relativizer (classifier (CL) versus relative marker *ge3* RCs). Accuracy measures and looking preference to the target were analyzed using generalized linear mixed effects models. Results indicated Cantonese children with DLD scored significantly lower than their AM-TD peers in accuracy and processed RCs significantly slower than AM-TDs, but did not differ from the YTDs on either measure. Overall, while the results revealed evidence of a SRC advantage in the accuracy data, there was no indication of additional difficulty associated with ORCs in the eye-tracking data. All children showed a processing advantage for the frequent CL relativizer over the less frequent *ge3* relativizer. These findings pose challenges to domain-specific representational deficit accounts of DLD, which primarily explain the disorder as a syntactic deficit, and are better explained by domain-general accounts that explain acquisition and processing as emergent properties of multiple converging linguistic and non-linguistic processes.

## Introduction

Developmental Language Disorder (DLD) is an impairment that primarily affects linguistic abilities in childhood, independent of any obvious accompanying conditions such as hearing loss, emotional and behavioral problems, intellectual disability and neurological problems. DLD is estimated to affect 7–11% of the population in English-speaking countries [1, 2], but does not affect all aspects of language equally. A notable weakness in DLD is in the production and comprehension of complex sentences. One specific structure commonly assessed is the relative clause (RC), in which children with DLD robustly perform below their typically-developing (TD) peers, a finding that has been observed across many different languages, including English [3], Danish [4], Greek [5], and Hebrew [6].

Studies investigating knowledge of RCs in children with DLD have focused on the relative ease of comprehending subject RCs (SRCs) and object RCs (ORCs) (also extensively studied in TD children and adults). Consider sentences (1) and (2), a subject and object RC, respectively.

1. The chicken that kissed the mouse.
2. The mouse that the chicken kissed.

Sentence (1) contains a SRC, where the head noun *chicken* serves as the subject of the RC *that kissed the mouse*. In contrast, (2) contains an ORC, where the head noun *mouse* serves as the object of the RC *that the chicken kissed*. The majority of the literature comparing SRCs versus ORCs in child language investigates English and other European languages, with results consistently indicating that SRCs are easier to acquire or process than ORCs in TD children (the so-called SRC (over ORC) advantage, or subject-(over-)object asymmetry; e.g. [7] in English and German; [8] in Italian, see also [9] in Hebrew, a Semitic language) and that DLD children performed significantly poorer than their TD peers and showed greater SRC over ORC advantage/asymmetry (e.g. [3] in English; [5] in Greek; [6] in Hebrew; [4] in Danish). However, this large skew in empirical base towards English and other mostly European languages in the RC acquisition literature means that the field lacks a data from a sufficiently diverse set of typologically different languages [10].

Notably, these well-studied languages contain postnominal RCs, in which the RC follows the noun it modifies. More recently, studies have focused on RC acquisition in East Asian languages, many of which have prenominal RCs. In contrast to the work on languages with post-nominal RCs, which attests a robust SRC advantage, acquisition studies in languages like Japanese [11, 12], Mandarin [13] and Cantonese [14, 15] paint a more complex picture, with studies frequently reporting either an ORC advantage or no difference. It is therefore important to study typologically diverse languages to examine how language-specific effects manifest in acquisition. In the following sections, we first introduce the prominent theoretical explanations for subject-object asymmetry in the RC literature, followed by an overview of the domain-specific versus domain-general accounts of acquisition and the nature of difficulties in and predictions for children with DLD, and then move onto discussing the RC acquisition and processing issues and studies in Cantonese, the language under current investigation.

### Theoretical explanations for subject-object asymmetry

Central to the theoretical discussion of the source of subject-object asymmetry in the RC literature is resolving the long-distance dependencies, the process in which the parser must integrate information from the head noun (filler) to the so-called gap (that has a conceivable missing argument) to interpret the RC. See (1) repeated here as an illustration of the location of the head noun and the gap.

(1) [_head noun_ The chicken_i_] [_RC_ that ___i_ kissed the mouse].

Prominent theories of RC acquisition and processing differ in terms of their characterization of the processing asymmetry as arising from memory / cognitive resources, structural factors, prominence effects and experience-based effects. This section discusses each of these factors in turn.

### Memory/ Resource-based effects: Linear distance factor

**Fig 1:**
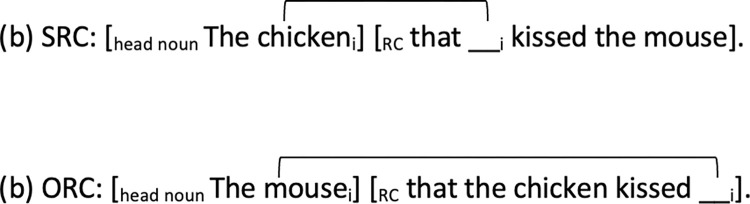
Examples of English SRC and ORC with linear distance between the filler and the gap indicated.

One set of proposals concern the cognitive load in processing the dependency, which is computed by calculating the linear filler-gap distance (i.e. the number of intervening elements) between the RC head noun (filler) and the gap on the surface form. The assumption is that the parser must retain information of the filler and other intervening elements in working memory until the gap position is encountered; thus, the longer linear distance the greater burden on working memory. While earlier proposals calculate linear distance in terms of the number of intervening words (e.g. [16]), Gibson’s Dependency Locality Theory [17, 18] considers the discourse-pragmatic properties on cognitive load and calculates processing cost in terms of the number of intervening new discourse referents denoted by noun phrases and verbs, because the integration and storage of such new information are additionally taxing on working memory (c.f. O’Grady [19] which also includes the linear distance factor in his processing-based account of RCs). In English, the linear distance factor favors the processing of SRCs over ORCs, because English ORC has a longer linear distance than SRC. See (1b) and (2b) Fig 1 below.

However, the linear distance-based prediction is dependent on the language under examination, specifically the word order configurations or head-directionality of the RCs, which can lead to different outcomes between languages. This will be further elaborated in our later discussion of Cantonese RCs.

### Structural factors: Hierarchical sentence structure, structural distance and structural intervention

Another way of computing the processing cost in formal or structural approaches is to consider the hierarchical sentence structure and the depth or embeddedness of a gap. The core idea is that higher processing cost is associated with constituents that are more deeply embedded (hence more difficult to access) in hierarchical structure. Among the various metrics proposed to determine such structural effects, one (earlier) approach is to measure the structural distance between the filler and the gap position, as represented by the embeddedness of the gap in terms of intervening syntactic nodes in a hierarchical structure [20–22]: the longer the structural distance between the filler and the gap, the more taxing it would be to process the dependency. As shown by the hierarchical representations of the English paired examples (1c & 2c) below, English ORCs are more deeply embedded and have a longer structural distance between the filler and the gap and are thus predicted to cause more difficulties than SRCs.

**Fig 2:**
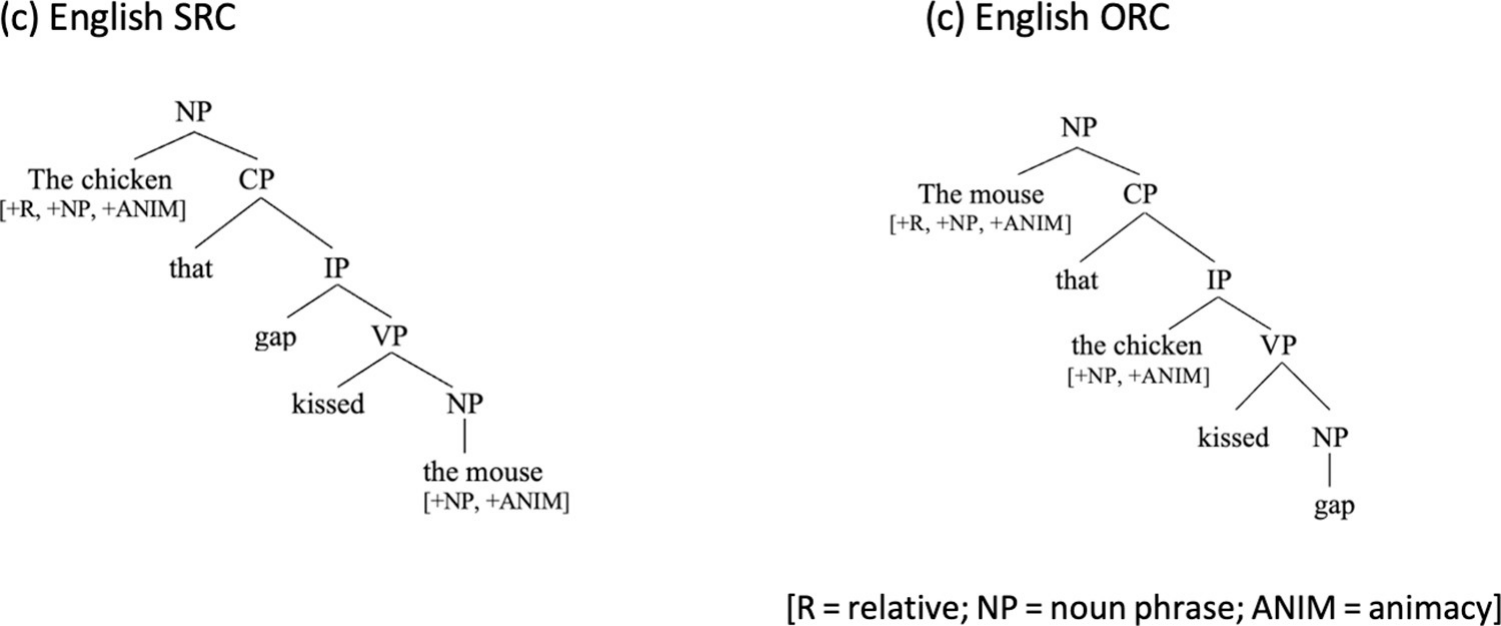
Hierarchical structure of English SRC and ORC.

One structurally-oriented approach that has had a strong influence even in the current RC literature involves the notion of Relativized Minimality [9]. According to this approach, a dependency is more difficult to process when there is a structural intervener between the filler and the gap, violating Relativized Minimality [23, 24]. In English ORCs, as in (2c) in (Fig 2), the dependency between the head/filler (“the mouse”) and the gap must cross over the RC-internal subject (“the chicken”), with which it shares a subset of identical formal features. As such, the RC-internal subject becomes a structural intervener blocking the local relation between the head/filler and the gap, violating Relativized Minimality. By contrast, no structural intervener occurs between the head/filler and the gap in English SRCs like (1c) in (Fig 1). Thus, SRCs are predicted to be easier to process/acquire.

Because in nominative-accusative languages subjects unambiguously hold a higher position than objects in structural representations, structural effects support a subject-over-object advantage regardless of cross-linguistic variations in RC surface configurations, unlike linear distance-based accounts (see [25] for an in-depth review).

### General subject prominence effect

The prominence of subjects in discourse is regarded as another factor that is of special relevance to RC acquisition and processing [19]. The notion of general subject prominence builds on the functional notions of topicality and foregrounding [26–28] and relates to resolving an ‘aboutness’ relationship between RC and the head noun [26]: a RC is functionally about the referent of the head noun and there is a general facilitating effect from subject prominence in the construal of such an aboutness relationship, because grammatical subjects are often the default topics and therefore are typically highly salient in discourse. As such, it is easier to process a RC that describes the subject rather than the object, which is less accessible and less prominent in discourse.

Focusing on the functional discourse properties of subjects as default discourse topics, the general subject prominence effect is not subject to crosslinguistic variations of RCs in surface configurations and is expected to be present across languages, especially in nominative-accusative languages (c.f. [25]). However, its influence may be moderated by interacting factors, such as experience [19].

### Experience-based effects: Frequency, canonical structures, and relationship between constructions

Another set of explanations for subject-object asymmetry in the RC literature considers the role of learner’s language-specific experience. The basic idea is that experience-based frequency effects are expected in acquisition [29], because the more frequently a word/pattern is experienced, the stronger its representation and the more accessible, thus easier, the corresponding processing becomes, as it meets the parser’s expectation of the upcoming elements [19, 30–33].

Frequency effects are closely related to influence from canonical word order and simpler, related constructions observed in acquisition (e.g. [7, 34]). The Canonical Word Order Hypothesis [34] concerns the surface similarity of any syntactic structures to the canonical word order configurations of the target language, suggesting that children tend to apply schemas of canonical sentences to help interpret other structures. As a SVO language, English has frequently occurring NVN exemplars that invoke a processing advantage for SRCs but not for ORCs, which deviate from the canonical word order schema. See the illustrated examples (1d & 2d) below.

N/S V N/O

(1d) SRC: [_head noun_ The chicken_i_] [_RC_ that ___i_ kissed the mouse].
N/O N/S V
(2d) ORC: [_head noun_ The mouse_i_] [_RC_ that the chicken kissed ___i_].

Beyond canonical word order, more developmental evidence points to a facilitating effect of similarity with simpler known constructions in the acquisition of complex structures like RCs (e.g. [7, 35] for English RCs; [36] for East Asian RCs; see also [13, 37] for Chinese RCs). The idea is consistent with the ‘construction conspiracy hypothesis’ [38], which proposes that the acquisition of new, complex constructions is supported by prior acquisition of simpler, related constructions with overlapping form and/or function (see similar proposal by [39]). Consistent with the hypothesis, the authors demonstrated that the acquisition of the *sein*- passive by a German-speaking boy was supported by simpler, early-acquired *sein* copular construction (as a source construction), while this was not observed for the *werden*-passive. In relation to RCs, Diessel and Tomasello [7] argued that it is the frequent occurrence of the agent being expressed by the sentence-initial NP in the target language, rather than a fully developed word order schema that accounts for the SRC over ORC advantage in English- and German- speaking children’s RC production. Similarly, Fitz et al.’s connectionist model [40] provided computational evidence that the SRC or over ORC advantage in English could be explained by the more frequently experienced SRC substructure of “THAT VERB” than the ORC substructure of “THAT ARTICLE NOUN” in the input.

As such, relationships between constructions implicate structural frequency in a learner’s language-specific experience. Within an interrelated network of constructions [41], frequency effects can co-exist at different levels of granularity, ranging from the target structures which may involve concrete lexical strings, to sequences that resemble the target structures, to abstract cues such as word order properties configurations [29]. In other words, experience-based frequency effects are indexed by not only the target construction but also its related constructions at a more general level defined by common mappings from linear order to functional roles [42, 43].

### Domain-specific versus domain-general accounts of acquisition: Subject versus object RCs

Theories of language acquisition differ in their characterization of the mechanisms that support language learning. Domain-specific approaches assume a specialized module devoted specifically to language, arguing that the acquisition of morphosyntax involves structural principles and constraints which primarily guide language acquisition. Among the RC theories discussed in the previous section, formal approaches concerning effects of structural complexity in terms of structural distance or structural intervention on hierarchical sentence structures can therefore be categorized under domain-specific accounts of acquisition (e.g. [9, 23, 24]). Given that subjects constitute a higher position than objects in hierarchical representations in most languages, domain-specific structural perspectives uniformly predict a SRC over ORC advantage.

By contrast, domain-general accounts of language acquisition view language as an integral facet of cognition and posit that general cognitive mechanisms that subserve all kinds of learning, not just language, support language acquisition. A prominent domain-general account, the emergentist approach to language acquisition, views acquisition outcomes as emerging from the interaction or even competition of multiple general factors that could vary in strength across languages or across time in a child’s development, giving primacy to general factors like learner’s experience, reliability of form-meaning mappings, cognitive abilities, and processing [44]. As such, unlike domain-specific approaches that have a uniform prediction on subject-object asymmetry based on structural factors (e.g. a SRC-over-ORC advantage in Hebrew [9, 45], Italian [45, 46], Chinese [47, 48] and see also [25] for a review of SRC preference predicted for other nominative-accusative languages, based on structural constraints associated with hierarchical sentence structures), variations in acquisition outcomes are expected and constitute a core conceptual theme in domain-general emergentist approaches.

Accounting for RC acquisition from an emergentist perspective, learner’s language-specific experience plays a core role in shaping processing routines and acquisition outcomes [19, 30, 31, 49]. Thus, in the RC theories discussed, experience-based frequency effects including support from simpler, known constructions are directly applicable within the domain-general framework (e.g. [7, 14, 40]). O’Grady’s emergentist approach [19] highlights two additional relevant factors among the RC acquisition/processing theories discussed, namely, general subject prominence and the linear distance effects, that interact to contribute to processing cost While the existence of hierarchical representations is assumed in the Dependency Locality Theory [17, 18], the domain-general emergentist account does not make such an assumption but rather considers the linear distance effects (in terms of intervening elements) as simply postponing the resolution of filler-gap dependency, which invokes greater working memory burden [19]. Therefore, in English, experience-based frequency, general subject prominence and linear distance effects all conspire to create a strong SRC advantage. However, as the next section on Cantonese RCs will illustrate, domain-general predictions can vary across languages according to differences in surface word order configurations and variations in form-function overlaps in the target language.

### Domain-specific versus domain-general accounts of acquisition: Predictions on DLD versus TD

Domain-specific, structurally-oriented perspective explains grammatical impairments in DLD as an outcome arising from deficits in grammatical computations. Specifically, domain-specific accounts of DLD such as the Computational Grammatical Complexity (CGC) account [50] and the Edge feature Underspecification Deficit [51, 52] propose that children with DLD have a core, representational deficit that affects all syntactic dependencies derived by movement, which include RCs. According to van der Lely [50], ‘a core deficit will be significantly below age-matched peers’ performance and often below other language abilities: for example, grammatically-impaired children perform significantly worse on tasks that tap aspects of morpho-syntax than younger children matched on vocabulary, or on general measures of grammar’ (p.54). As such, the domain-specific representational deficit accounts of DLD predict that children with DLD have a specific difficulty with all movement related structures including RCs (i.e., more than a general language delay), scoring not only lower than their age-matched typically-developing (AM-TD) peers but also younger, language-matched typically-developing (YTD) children in RC competence.

By contrast, domain-general accounts consider the language deficits in DLD as arising from basic cognitive processes that support learning in general. For instance, the limited processing capacity accounts [53] suggest that children with DLD have general processing deficits affecting phonological or working memory limitations that result in reduced processing speed. Other cognitively-oriented approaches also suggest weaker statistical learning abilities in DLD children (e.g. [54–56]), which could affect their uptake of linguistic input and account for their difficulties in language development. On this account, DLD arises from children’s limited ability to process and extract regularities from the input, predicting a global language delay rather than a specific grammatical deficit with movement-related constructions including RCs [57]. Thus, although children with DLD would score lower than their AM-TD peers when being assessed on their RC competence, they are not expected to score even worse than their younger, language-matched children in RC competence (as an illustration of exhibiting a specific difficulty with RCs that is more than a general language delay).

### The current study and predictions on Cantonese

Chinese languages like Cantonese and Mandarin attest a typologically rare combination of SVO word order with relative clauses preceding the head noun [58, 59], with this unique word order property resulting in some processing factors favoring subject RCs while others favoring object RCs [37]. While Cantonese and Mandarin RCs are similar in word order configurations, there are characteristics unique to Cantonese RCs that further impact processing demands (see [14, 37] for a discussion of similarities and differences between Cantonese and Mandarin). The current study focused on Cantonese RCs and aimed to test the contrasting theoretical predictions of the domain-specific versus domain-general approaches to explaining RC acquisition and processing in Cantonese-speaking children with and without DLD.

**Fig 3:**
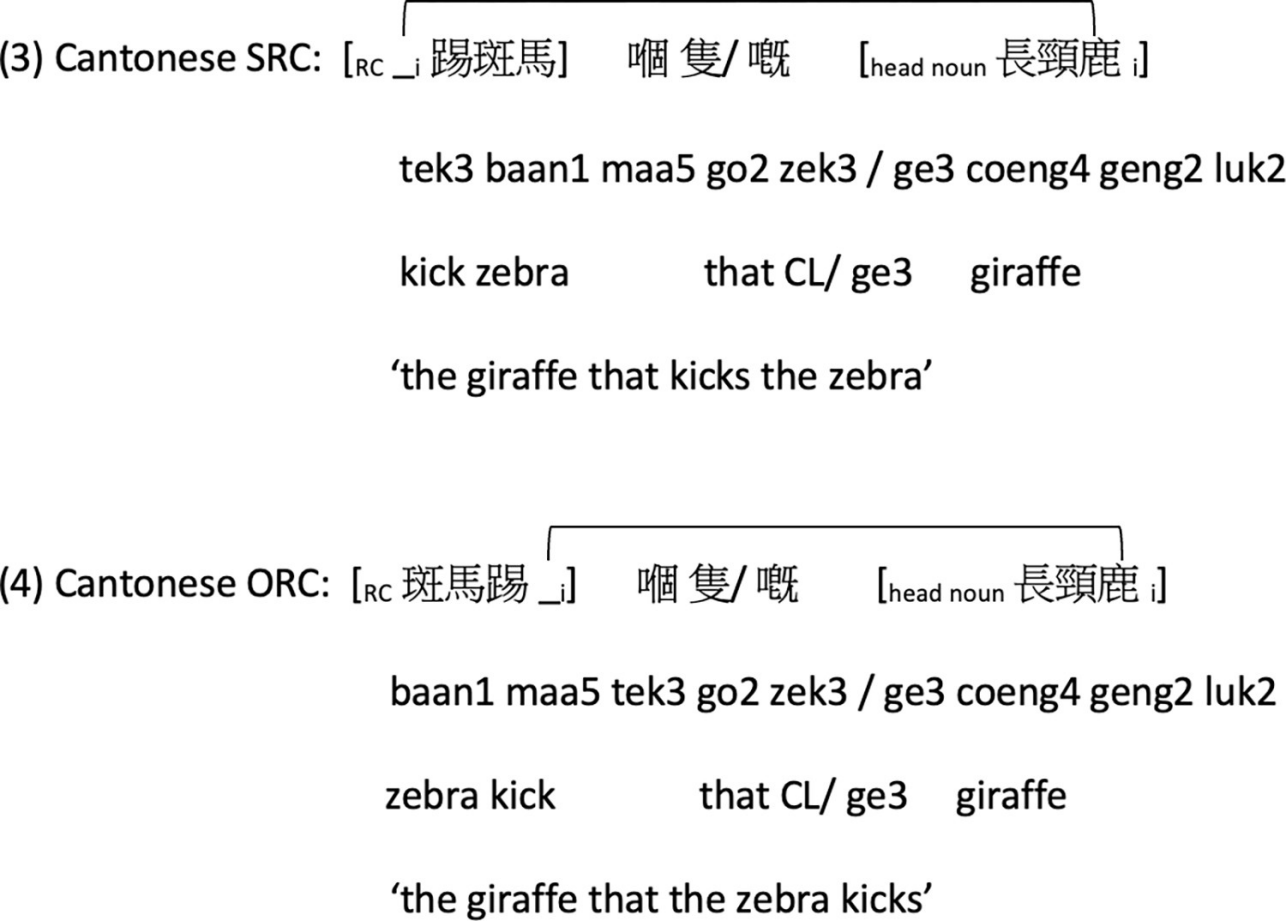
Examples of Cantonese SRC and ORC with linear distance between the filler and the gap indicated.

As mentioned above, Cantonese relative clauses are prenominal because they are placed before the head noun. There are two relativizers in Cantonese: classifier (CL) and the particle *ge3* RCs, as shown in (3) and (4) in Fig 3.

**Fig 4:**
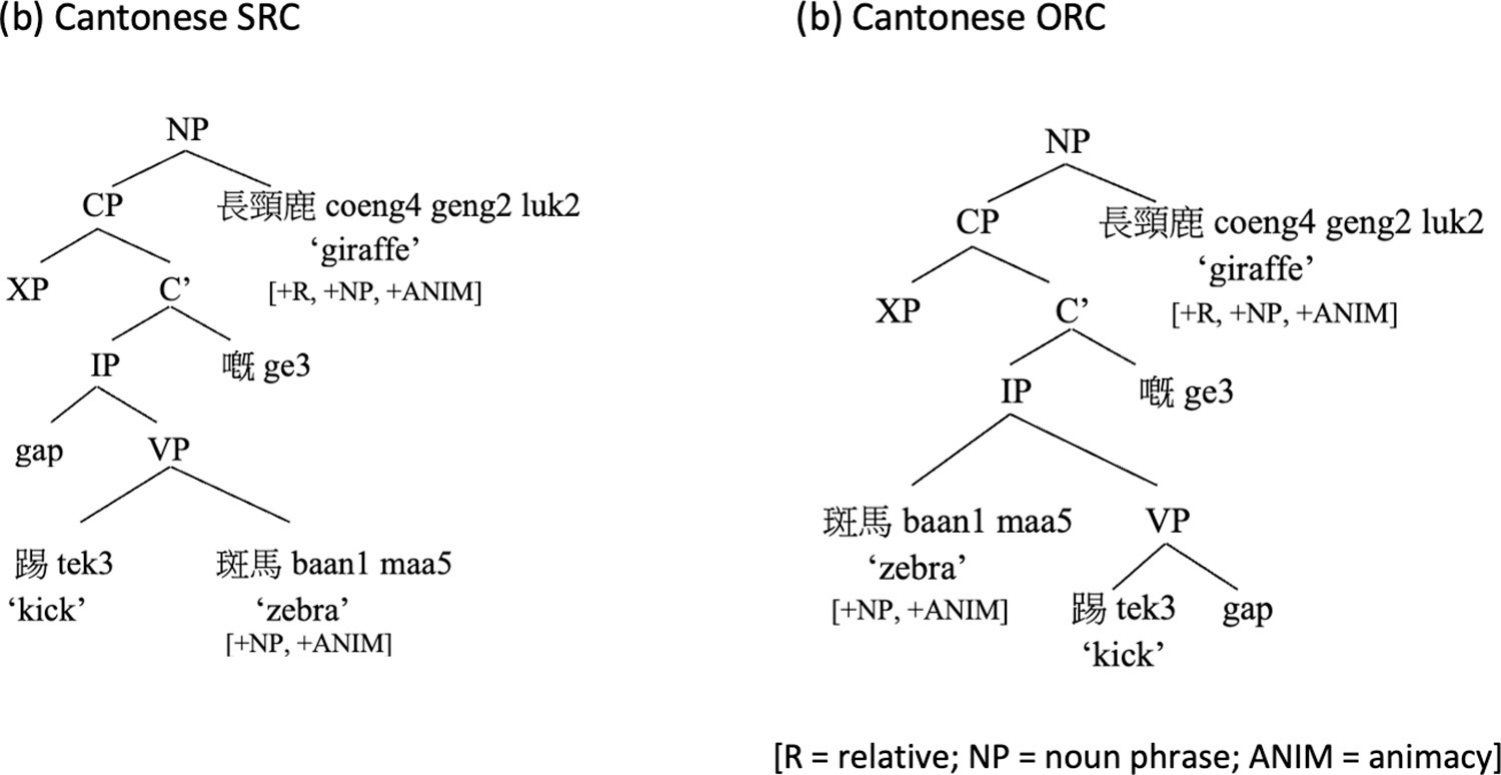
Hierarchical structure of Cantonese SRC and ORC.

These typological properties of Cantonese present a unique opportunity to tease apart the predictions of domain-specific and domain-general approaches on subject-object asymmetry in a way that is not possible in past studies on other languages with post-nominal RCs. Consider the structural representations of Cantonese SRC and ORC assumed in the domain-specific approaches, as shown in (3b) and (4b) in Fig 4. The filler-gap dependency in ORC is intervened by the RC-internal subject; whereas there is no structural intervener in SRC in the hierarchical structure. Structural intervention accounts therefore predict a SRC-over-ORC advantage in Cantonese (see [47, 48] for similar predictions in Mandarin).

On the other hand, factors in domain-general approaches no longer coalesce to create a strong SRC-over-ORC preference in Cantonese. While *prominence* predicts a general SRC advantage across languages, a shorter linear filler-gap distance favors ORCs in Cantonese (see [60] for similar predictions in Mandarin), as illustrated in (3) and (4). Moreover, Cantonese ORCs (not SRCs) resemble simple SVO transitive constructions in the language ([15, 37]; see also [36] and [13] for similar account of Mandarin RCs), and hence their processing may be supported by children’s knowledge of this simpler, frequent, and early acquired construction. Overall, the three factors of subject prominence, filler-gap linear distance, and experience-based frequency effects pull in opposite directions in Cantonese. Depending on which factor is stronger, domain-general approaches predict at least a lack of SRC advantage (if not an ORC advantage) or only a weak SRC advantage (if any) when these factors interact.

Regarding predictions on DLD versus TD children, domain-specific and domain-general accounts also make different predictions. Recall domain-specific representational deficits accounts [50–52] predict that children with DLD would perform worse than both their age-matched and younger, language-matched TD peers, displaying a specific difficulty with long dependencies, movement-related structures like RCs, that is more than a general language delay. In contrast, the domain-general approach would predict a global language delay (not a specific difficulty with RCs) in children with DLD, where their performance could resemble the younger, language-matched TD children but worse than their age-matched peers.

Furthermore, a specific prediction about the two relativizers (CL vs *ge3*) in Cantonese can be formed under the domain-general framework, which emphasizes experience-based effects in acquisition. Belonging to different functional registers, CL RCs are used more often in colloquial speech, while *ge3* RCs are often used in formal registers like news reporting and literary texts [37, 61]. CL RCs are therefore more frequently experienced by younger children in child-directed language, while *ge3* RCs become more frequently encountered when children grow older and gain more experience with formal speech registers and text. As such, domain-general accounts predict a general CL over *ge3* advantage. In contrast, domain-specific perspectives make no explicit predictions regarding frequency effects in processing/acquisition, as they are considered peripheral to core grammar; see also works such as [62, 63] for a review which may be considered an exception.

The differing predictions of the two approaches, as outlined above, are summarised in Table 1.

**Table 1:** Predictions of domain-specific versus domain-general accounts for the acquisition and processing of Cantonese RCs.

	Domain-specific	Domain-general
**SRC vs ORC**	a uniform SRC over ORC advantage in Cantonese	a lack of SRC advantage (if not an ORC advantage) or only a weak SRC advantage (if any)
**CL vs *ge3***	No explicit prediction (as frequency information are peripheral to core grammar)	a CL over *ge3* advantage (as frequency effects favor CL RCs)
**DLD vs TD peers**	a specific difficulty with RCs in DLD (i.e. more than a general language delay): DLD < AM-TD; DLD <YTD	a global language delay in DLD (i.e. not a specific difficulty with RCs): DLD <AM-TD; DLD = YTD

DLD: Developmental Language Disorder; AM-TD: age-matched typically developing peers; YTD: younger language-matched typically developing peers

Turning to our current knowledge base on child language acquisition studies on Cantonese RCs, studies on typically-developing children are still relatively infrequent and existing evidence regarding the relative ease of SRCs vs ORCs is mixed. For instance, Yip and Matthews [15] reported that ORCs either emerged earlier or concurrently as SRCs in the three bilingual children’s naturalistic speech in Cantonese. Lau [64] reported a SRC-over-ORC advantage in a picture-based character identification task testing RC comprehension by children aged 3;0–5;11, but no SRC/ORC advantage in elicited production from children aged 4;0–5;10. However, Chan et al. [14] recently reported a robust ORC-over-SRC advantage using elicited production from Cantonese three-year-olds; while Chan et al. [65] found a significant ORC advantage in CL RCs but SRC advantage in *ge3* RCs in four-year-old Cantonese-speaking children’s online comprehension. Taken together, these mixed findings call for a theoretical account that can explain these variations observed.

As for the context of DLD, there has been no published research on the syntactic competence of RCs in Cantonese-speaking children with DLD. In fact, there are only a few published studies on identifying the linguistic features of DLD in Cantonese such as aspect markers, passives and wh-questions [66–68], even though syntactic complexity has been reported to best differentiate children with and without DLD in Cantonese [69]. Thus, the syntactic competence of RCs in Cantonese DLD children remains an issue that deserves more research attention. On the other hand, there is some work on RC comprehension [70] and RC production studies [51, 52] in Mandarin-speaking children with and without DLD. The three studies reported mixed findings of a significant SRC over ORC advantage in DLD children but a lack of subject/object advantage in TD children using a picture matching task [70]; a SRC advantage in both DLD and TD children using an elicited production task [51] but a lack of subject/object advantage in all children using an imitation task [52]. Consistent among these three recent Mandarin RC studies are the findings that Mandarin DLD children’s RC performance scored significantly lower than both their AM-TD peers and the YTD children, which the authors claimed as supporting evidence showing a specific difficulty with RCs that aligns with the representational deficits accounts of DLD [50–52].

However, the sampling of a YTD group across the three Mandarin DLD studies on RCs was not standard. In the DLD literature, it is typical to sample a TD group of at least two years younger (e.g. [3]) whose overall language competence should be comparable; but these three Mandarin DLD studies depart from the DLD literature conventions, in which the younger TD group was only one-year apart from the chronologically age-matched TDs and their standardized language test scores were also comparable or even numerically better than the AM-TDs. As such, when the children with DLD scored even lower than their YTDs in RC competence, it could be due to their YTDs having better overall language competence that is comparable to the AM-TDs, rather than a representational deficit in DLD children that specifically affects their RC performance as the authors claimed. To tease apart these factors, further investigation sampling a language-matched YTD group is therefore warranted.

Given the above theoretical background and literature review, the current study is a first attempt to examine RC comprehension in Cantonese-speaking children with and without DLD. We test the contrasting theoretical predictions of domain-specific versus domain-general accounts of Cantonese RCs and children with DLD across several dimensions. Children with DLD were compared with their age-matched TD (AM-TD) and language-matched (and therefore younger) TD peers (YTD; c.f. [3]). Specifically, we examined children’s performance on two RC types (SRCs versus ORCs) and two relativizers (CL versus *ge3*).

## Method

### Participants

Sixty-eight predominantly monolingual Cantonese-speaking children were recruited from schools in Hong Kong to participate in this study. The current study was approved by the Human Subjects Ethics Sub-committee at the Hong Kong Polytechnic University (approval number: HSEARS20161230004). Informed written consent to participate in this study was obtained from the participants’ legal guardians or next of kin. All participants were assessed by speech therapists, passed hearing screening, and completed the standardized norm-referenced language tests to confirm their clinical status (Hong Kong Cantonese Oral Language Assessment Scale (HKCOLAS, [71]) for school-aged children; or the Cantonese version of the Reynell Developmental Language Scales (RDLS-R and RDLS-E, [72] for preschool children). They were attending local mainstream primary schools or kindergartens using Cantonese as the medium of instruction, receiving the same regular education despite their language status. Twenty-three children were identified as DLD based on Bishop et al. [73]’s diagnostic recommendations: (i) the children scored 1.25 or greater SDs below age means in two or more out of six subtests of the norm-referenced Hong Kong Cantonese Oral Language Assessment Scale (HKCOLAS; [71]) in their L1 Cantonese); (ii) their language difficulties had negative functional impact affecting daily social interactions or educational progress based on parental and/or school expressed concerns; (iii) there were poor prognostic features such as difficulties affecting multiple areas of language functioning, including receptive language and language learning difficulties persisting till aged 5 or above; and (iv) there was absence of associated biomedical conditions such as absence of hearing disability, intellectual disability or another diagnosis of neurodiversity (e.g., Autism Spectrum Disorder). Each DLD child was individually matched to a typically-developing child according to age (+ or– 4 months) and grade, and as such both DLDs (N = 22) and AM-TDs (N = 23) were aged between 6;6–9;7. One child with DLD was excluded because his data were uncodable due to technical issues during data collection. In addition, we included a group of younger and language-matched typically-developing children (N = 21; aged between 4;7 and 7;6), with each child being about two years younger than a corresponding DLD child [3]. One YTD participant was excluded because she did not attend all the experimental sessions.

The younger group of typically developing children (YTD; HKCOLAS: *M* = 196.94, *SD* = 62.11; Receptive Grammar: *M* = 39.56, *SD* = 9.32) did not differ from DLD group (HKCOLAS: *M* = 180.85, *SD* = 48.92; Receptive Grammar: *M* = 35.60, *SD* = 6.85) in their overall language scores in general, *t*(34) = -0.87, *p* = .390 and their subtest scores on receptive grammar in particular in HKCOLAS, *t*(34) = -1.47, *p* = .151. Five YTD and two DLD participants were excluded from this comparison because of the following reasons: (i) these 5 YTD children were below age 5 at the time of testing and were administered the Cantonese version of Reynell Developmental Language Scales instead of HKCOLAS which is intended for children aged 5 to 12 (and therefore while we could confirm their TD status, their HKCOLAS scores were not available for direct comparisons with other children); (ii) 2 DLD children did not meet the inclusionary criteria for our data analyses (see Results section for details) and therefore were subsequently excluded.

### Materials and tasks

#### Language assessments

Children’s clinical language status was informed by their performance in HKCOLAS [71], a standardized norm-referenced language test that consists of six subtests: Test of Hong Kong Cantonese Grammar, Textual Comprehension Test, Word Definition Test, Lexical-Semantic Relations Test, Narrative Test and Expressive Nominal Vocabulary Test. Five participants from the YTD group were assessed by another standardized norm-referenced language assessment, the Cantonese version of Reynell Developmental Language Scales (RDLS-R and RDLS-E, [72]) that assessed verbal comprehension and expression, instead of HKCOLAS as they had not reached the minimum age of conducting HKCOLAS (i.e. 5 years old) at the time of testing.

#### Relative clause comprehension task

Sixteen experimental sentences, the same items as Chan et al. [65], were used in the eye-tracking comprehension task: eight CL and eight *ge3* relative clause constructions, with four subject-extracted and four object-extracted in each condition. Each sentence contained common animal names (*bear*, *cow*, *dog*, *elephant*, *giraffe*, *horse*, *lion*, *monkey*, *panda*, *pig*, *tiger*, *zebra*) and transitive action verbs (*bite*, *bump*, *chase*, *feed*, *kick*, *lick*, *push*, *tickle*, *wipe*), all of which are familiar to children. A native speaker of Cantonese pre-recorded these sentence stimuli. Relativizer (i.e. CL versus *ge3*) and RC type (subject versus object) were tested as within-participants variables. See S1 Appendix for a complete list of sentence stimuli.

### Experimental procedure

**Fig 5:**
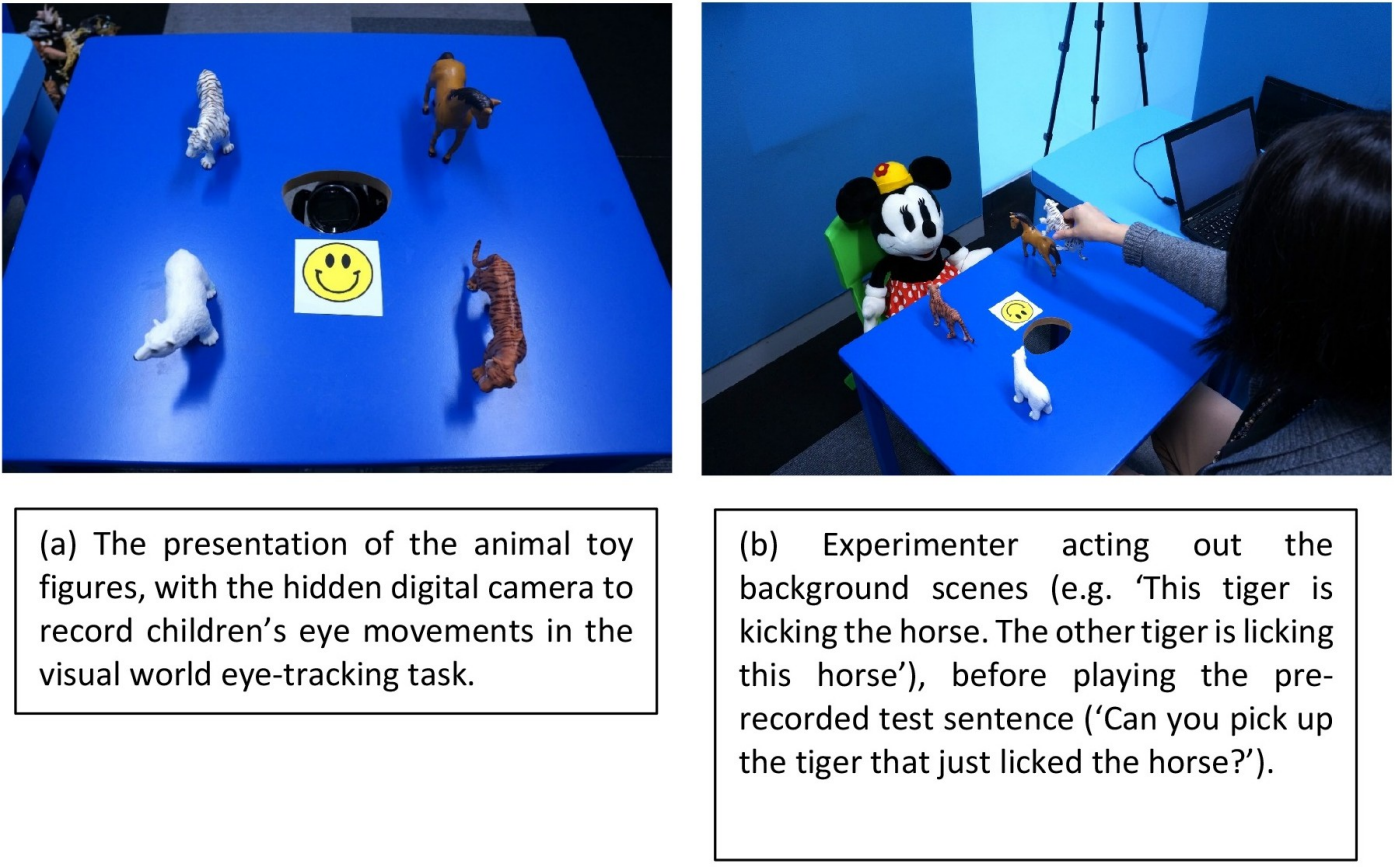
Experimental set-up.

We used the referent selection task in Chan et al. [65], which was adapted from Brandt, Kidd, Lieven and Tomasello’s [74]. In this task, children interact with an experimenter while they hear a range of sentences, some of which are the crucial RC test trials, and their eye-movements to toy referents are recorded. In each trial, four animals (i.e. target, distractor, related character, irrelevant character) are placed on the four corners of a table that has a hole cut at the center, allowing a central video camera to protrude from below and record children’s eye movements. Another camera was placed overhead, to record the entire experiment for cross-checking the accuracy data. There were two experimenters, one responsible for monitoring the camera to ensure children’s eye movements were recorded and for playing the prerecorded experimental items from a laptop; while the other experimenter was in charge of placing the toy referents at their pre-specified locations on the table and acting out the background scenes within an experimental trial. To ascertain whether children knew the names of the animal figures, the task began with the experimenter asking the child to name each toy on the table. In the rare cases when children provided a label that was different to our experimental stimuli, the experimenter corrected the child. See Fig 5 for the experimental set-up.

Previous studies have indicated a need to present a felicitous discourse context in RC processing studies [75, 76]. Following Chan et al. [65], we fulfilled this condition by creating two background scenes prior to playing the target sentence that contained a RC: one target scene as in (a) and one distractor scene as in (b). The animal toys were returned to their prespecified positions after each sentence was played and acted out by the experimenter. Before the target test sentence was played, we inserted an attention getter “Now look at the smiley face” as in (c) to divert the child’s eye gaze to the center, instead of looking to toy referents mentioned in the background scenes. The target test sentence was then played to the child, as in (d). A complete trial is included below, i.e. (a) to (d). There were four scripts, each containing a total of sixteen trials, but with a different random ordering of stimuli. The assignment of scripts was counterbalanced across children, with each child assigned to one of the four scripts. Across trials within a script, the order of presenting the background scenes was also counterbalanced: half presenting the target scene first, distractor scene the second, and vice-versa. The location of the toys was also pseudo-randomized across trials within a script, constrained by the requirement that the target head referent and the distractor being placed horizontally or diagonally from the child’s perspective, but never appearing on the same vertical plane where one was behind the other, which facilitated coding because it enabled blind coders to unambiguously know at which toy the child was looking. Children’s final choice of toy referent provided the accuracy data of their RC comprehension. The entire experiment lasted approximately 20 minutes for each child, with two practice trials included in the beginning to familiarize children with the requirements of the task.

(a) 睇吓!呢隻老虎踢緊呢隻馬仔喎
  tai2 haa2! ne1 zek3 lou5fu2 tek3-gan2 ne1 zek3 maa5zai2 wo3
  look PRT this CL tiger kick-PROG this CL horse SFP
  ‘Look! This tiger is kicking the horse.’
(b) 咦!另外一隻老虎就舐緊呢隻馬仔
  ji2! ling6 ngoi6 jat1 zek3 lou5fu2 zau6 lem2-gan2 ne1 zek3 maa5zai2
  EXCL another one CL tiger ADV lick-PROG this CL horse
  ‘The other tiger is licking this horse.’
(c) 而家, 睇下個哈哈笑公仔呀
  ji4 gaa1, tai2haa5 go3 haa1haa1siu3 gung1zai2 aa1
  now look at CL smiley figure SFP
  ‘Now look at the smiley face.’
(d) 你可唔可以拎起#頭先舐馬仔嘅老虎呀?
  nei5 ho2-m4-ho2ji5 ling1hei2 #tau4 sin1 lem2 maa5zai2 ge3 lou5fu2 aa3?
  you can-not-can pick up #just now lick horse ge3 tiger SFP
  ‘Can you pick up #the tiger that just licked the horse?’
  (#: pause)

### Comprehension accuracy scoring and eye-movement coding

Children’s final choice of toy referent (i.e. the toy that was picked up) served as a measure of their comprehension accuracy. A binary score of “0” was assigned for any incorrect response (i.e. toys other than the target referent) and “1” for a correct response. The scorings of 15% of the data were double-checked by a trained student helper and the agreement was 100%.

The camera placed under the table focused on the top-half part of children’s faces, allowing coding of their eye movements frame-by-frame to the four locations on the table using the visual editing program Sound Forge ©. This program displays the visual recording of the child’s face, with an audio track at the bottom, enabling researchers to select the critical time points of the target test sentence and code children’s eye movements frame by frame. Each frame was 40ms. Coding began at the onset of the first syllable of the RC, until 2400ms post RC-onset at 40ms intervals following the procedures reported in Chan et al. [65]. At each time frame, look to the target was coded as ‘1’; otherwise it was coded as ‘0’. Two experienced coders each coded approximately half of the dataset and their coding was further evaluated by a third experienced coder. Interrater reliabilities were high (coder A: 93.7%; coder B: 94.3%).

### Data analyses plan

To address the issue of subject-object asymmetry and whether RC comprehension is vulnerable in children with DLD, the first set of analyses focus on reporting comprehension accuracy, computed out of the total number of test items. In order to examine whether there is additional evidence of complexity effects captured by children’s eye-tracking data that could bear on the issue of subject-object asymmetry in RC comprehension, the second set of analyses report on children’s looking preference in their eye-tracking data based on the correctly interpreted test items (see results below for more details). The analyses of the looking behaviour thus asks a different question than the analyses of the comprehension data; namely, is there a *processing cost* associated with the successful comprehension of subject versus object RCs. This is the same analysis strategy adopted by Chan et al. [65] and Yang et al. [42].

## Results

Since one of our main goals was to examine children’s looking preference when they correctly interpreted an RC, children whose accuracy was too low for an accurate analysis of their eye movements were excluded from both comprehension accuracy and eye-tracking data analyses. The inclusion criterion was set to an overall 50% comprehension accuracy within a relativizer condition, following Chan et al. [65]. As such, four out of sixty-six children were further excluded for CL RCs; whereas eight out of sixty-six children were further excluded for *ge3* RCs. The final sample consisted of sixty-two children (19 DLDs (6;8–9;5, *M* = 7;8, *SD* = 0;8); 23 AM-TDs (6;6–9;7, *M* = 7;6, *SD* = 0;9); 20 YTDs (4;7–7;6, *M* = 5;7, *SD* = 0;9) for the CL condition and fifty-eight children (19 DLDs (6;8–9;5, *M* = 7;8, *SD* = 0;9); 23 AM-TDs (6;6–9;7, *M* = 7;6, *SD* = 0;9); 16 YTDs (4;8–7;6, *M* = 5;7, *SD* = 0;9)) for the *ge3* condition.

### Comprehension accuracy

**Fig 6:**
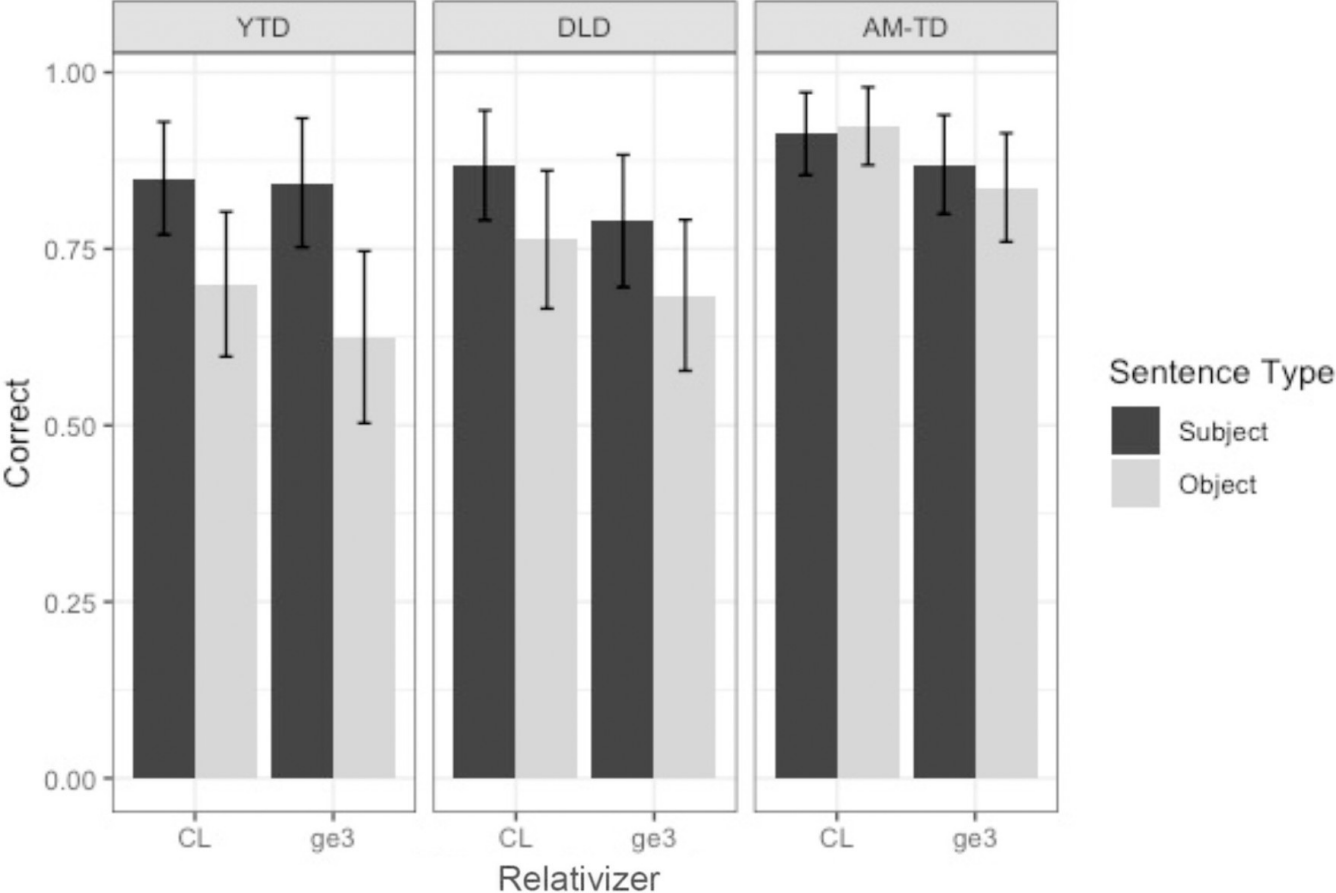
Comprehension accuracy of CL and *ge3* subject and object RCs by each group (error bars represent standard error).

The first set of analyses targeted children’s comprehension accuracy data by group (DLD, AM-TD, YTD), by relativizer (CL versus *ge3*) and by RC type (subject versus object). Fig 6 presents children’s comprehension accuracy of the CL and *ge3* subject and object RCs by the three groups of children (DLD versus AM-TD versus YTD).

As shown in Fig 6, all three groups of children performed better on CL RCs than *ge3* RCs. Within CL RCs, both DLD and YTD children scored higher on subject than object RCs whilst AM-TD group scored high on both object and subject RCs. For *ge3* RCs, all three groups of children did better on subject than object RCs. Children’s comprehension accuracy (correct = 1) was modelled using Generalized Linear Mixed Effects Models (GLMM; [77]) using the lme4 package for Linear Mixed Effects [78] in R (version 4.0.5; [79]). Relativizer (CL (0.5) versus *ge3* (-0.5); mean-centered), RC type (subject (0.5) versus object (-0.5); mean-centered), group (YTD versus DLD; DLD versus AM-TD; sliding contrast difference coding) and their interaction were entered as fixed effects. Random effects for participants and items were included [80].

Results from the mixed effects model are presented in Table 2. The significant main effects of RC type and relativizer indicated a significant subject over object and CL over *ge3* advantage for all children regardless of their groups. As predicted, DLD’s RC performance was significantly worse than their AM-TD peers across all conditions. On the other hand, the comparison between DLD and YTD children indicated no significant group difference. Further scrutiny of the error responses allowed us to better understand the underlying cause of the significant subject over object RC advantage. The error analyses revealed a frequent error type that was attested particularly prominently in DLD and YTD (not AM-TD) children when they comprehended ORCs (not SRCs): children made head noun assignment errors choosing the RC-internal subject (e.g. *zebra* instead of *giraffe* in (4)) erroneously as the head noun. The significant difference between SRCs versus ORCs therefore arose due to ORCs being mis-parsed as simple SVO sentences. We will discuss this point further in the discussion section.

**Table 2:** GLMM analysis summary for fixed effects predicting RC comprehension accuracy.

Fixed Effect	*Β*	*SE*	*z*	*P*
(Intercept)	1.61	0.13	12.29	<0.001***
RC Type (Subject)	0.61	0.21	2.87	<0.01**
Relativizer (CL)	0.48	0.21	2.25	<0.05*
Group (YTD vs DLD)	0.12	0.27	0.43	0.66
Group (DLD vs AM-TD)	0.86	0.28	3.04	<0.01**
RC Type (Subject): Relativizer (CL)	-0.18	0.42	-0.44	0.66
RC Type (Subject): Group (YTD vs DLD)	-0.42	0.42	-1.01	0.31
RC Type (Subject): Group (DLD vs AM-TD)	-0.61	0.45	-1.35	0.18
Relativizer (CL): Group (YTD vs DLD)	0.28	0.43	0.66	0.51
Relativizer (CL): Group (DLD vs AM-TD)	0.16	0.45	0.35	0.73
RC Type (Subject): Relativizer (CL): Group (YTD vs DLD)	0.48	0.83	0.57	0.57
RC Type (Subject): Relativizer (CL): Group (DLD vs AM-TD)	-0.59	0.90	-0.66	0.51

### Eye-tracking data: Looking preference

**Fig 7:**
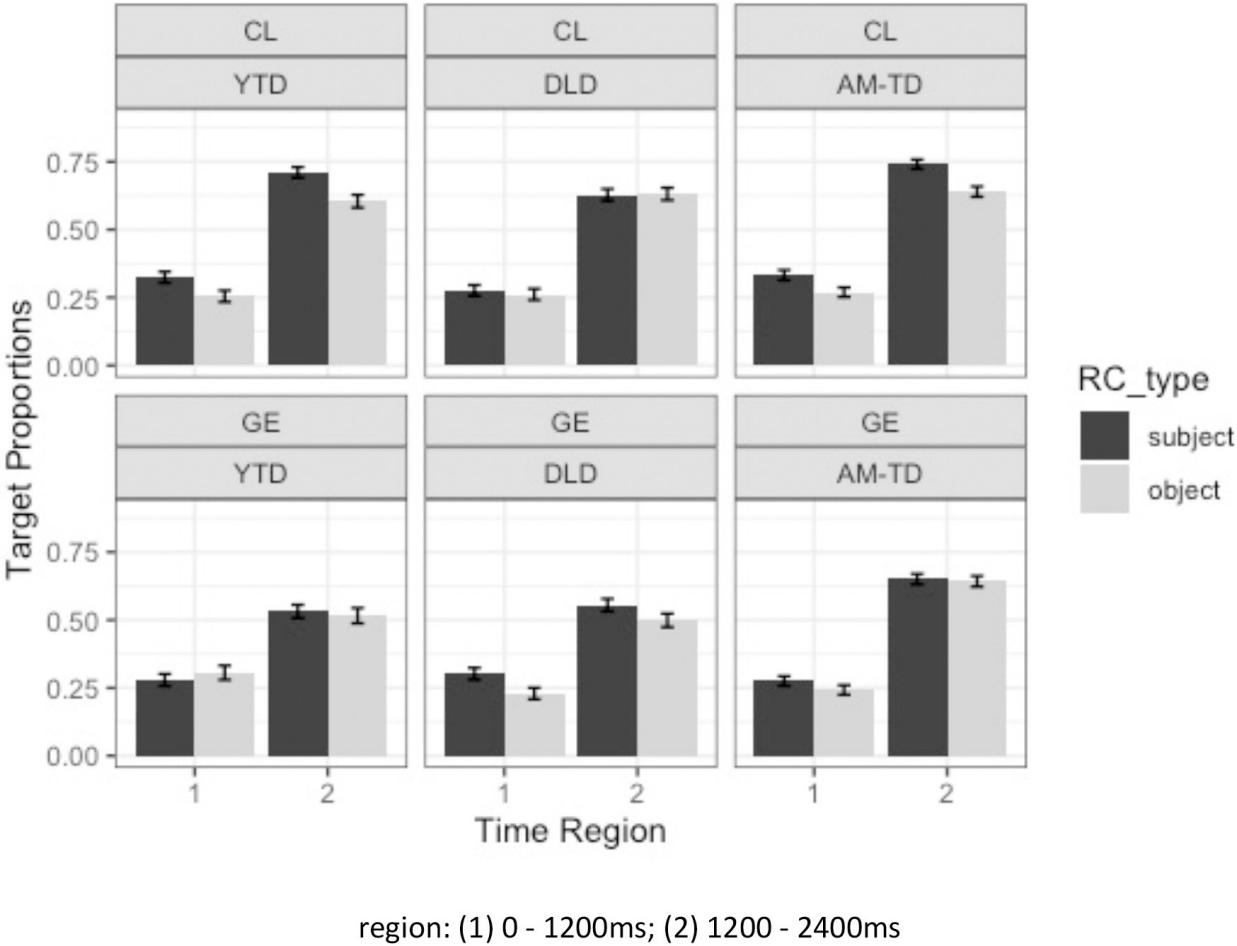
Average target proportions of looks for CL and *ge3* subject and object RCs in DLD, AM-TD, and YTD groups (error bars represent standard error).

The eye-tracking data analyses were modelled on preferential looking paradigms [81, 82]. The assumption is that children’s looking behaviors after listening to the auditory stimulus would reflect their comprehension: in a correct interpretation of a RC stimulus, children would show a significant increase to the target toy referent (that refers to the head noun of the RC heard), after hearing the head noun as the point of disambiguation for a prenominal RC, relative to their baseline looking preference before hearing the head noun. Fig 7 shows the average proportions of looks to the target across participants and items for CL and *ge3* subject and object RCs in DLD, AM-TD, and YTD groups.

To capture their processing differences over time, the time variable was divided into two regions from the RC onset to 2400ms post RC-onset (i.e. the first half from 0-1200ms and the latter from 1200-2400ms). Since Cantonese RCs are head-final, and the disambiguation point (head noun) started around 900ms (onset) to 1500ms (offset) after the RC onset, these two regions represent two distinct temporal phases: the first half (T1) mostly features processing before the head noun; while the second half (T2) mostly features processing after the head noun (i.e., when the identity of the head noun is clear). Since our eye tracking analyses focused on correctly interpreted test items, we would expect a significant increase in proportion of target looks in T2 relative to T1 (i.e. a significant main effect of time region), as evidence of children displaying their correct understanding of the RCs. Moreover, we also examined whether time region would interact with other variables of interest, to see whether the effect of time region was uniform or not across RC type (SRCs vs ORCs), relativizer (CL vs *ge3*) and our participant group (DLD vs AM-TD vs YTD). For instance, a significant interaction signaling that there was a higher increase in proportion of target looks from T1 to T2 in one condition than the other over the same time duration implies faster speed of processing and higher certainty with which participants converged on the correct token of the head referent across the crucial point of disambiguation. These possible interactions therefore allow us to capture differential speed of processing and certainty (if any) between RC type (SRCs vs ORCs), relativizer (CL vs *ge3*) and our participant group (DLD vs AM-TD vs YTD).

**Fig 8:**
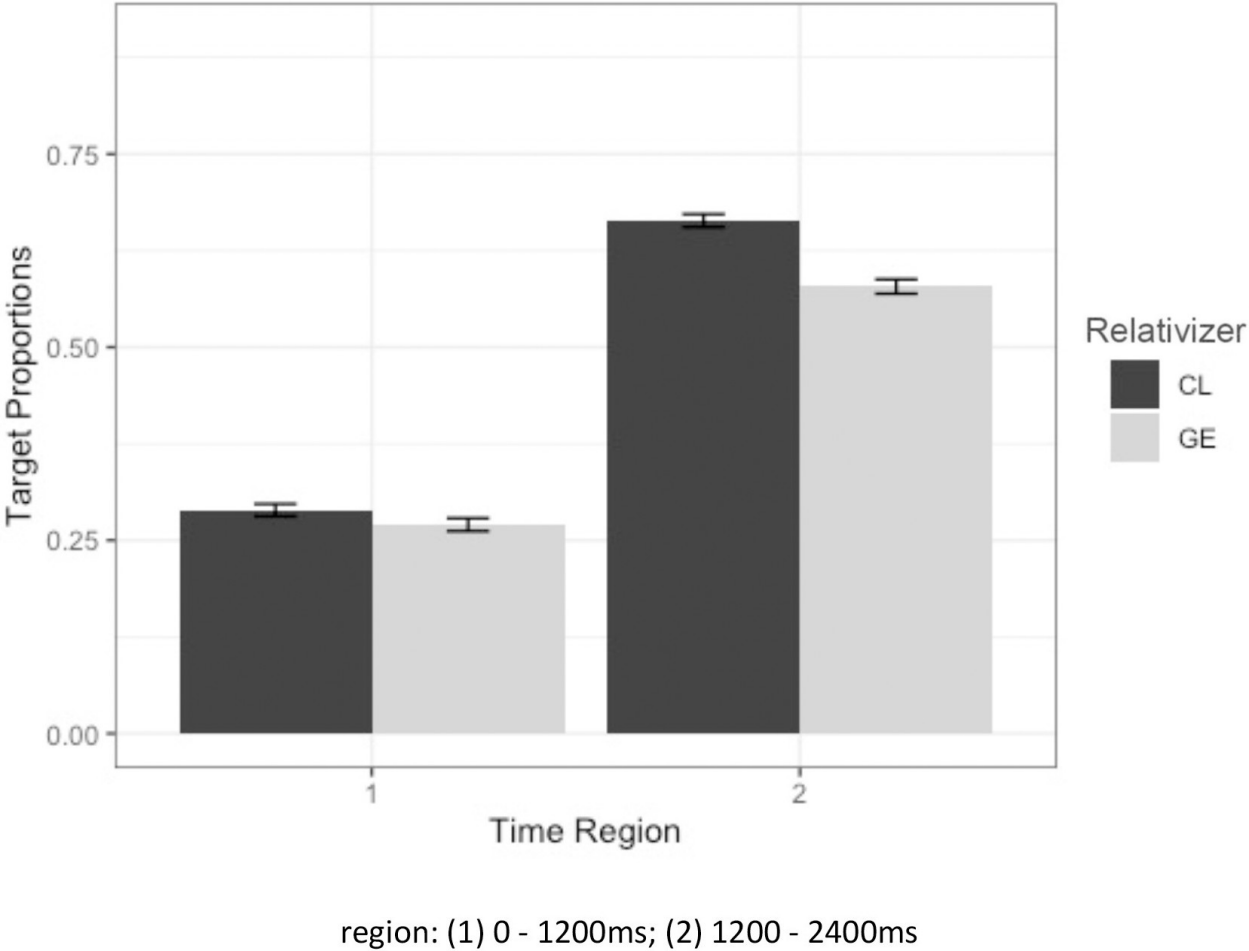
Average target proportions of looks for CL and *ge3* RCs in all children (error bars represent standard error).

Children’s looking preference to the target toy was predicted by Generalized Linear Mixed Effects Models (GLMM; [77]) using the *lme4* package for Linear Mixed Effects [78] in R (version 4.0.5; [79]). Relativizer (CL (0.5) versus *ge3* (-0.5); mean-centered), RC type (subject (0.5) versus object (-0.5); mean-centered), time region (1^st^ cluster versus 2^nd^ cluster) and group (YTD versus DLD; DLD versus AM-TD; sliding contrast difference coding) and their interactions were entered as fixed effects. Random effects for participants and random effects of items with the random slope of group were included [80]. As presented in Table 3, results from the mixed effects model showed a significant main effect of relativizer, where the proportions of target looks were higher in CL than *ge3* RCs, a significant effect of time region where the proportions of target looks were higher in the latter half of the time region (i.e. 2^nd^ cluster: 1200-2400ms) showing that, as expected, the children were converging on the target after they heard the head noun. There was no significant main effect of RC type nor of group. There was a significant two-way interaction between relativizer and time region, indicating that the increase of target looks across time was greater in the CL RCs condition than in the *ge3* RC condition, as can be seen in Fig 8, which plots the interaction.

**Table 3:** GLMM analysis summary for fixed effects predicting proportions of looks to the target in RC processing.

Fixed Effect	*Β*	*SE*	*z*	*P*
(Intercept)	-0.26	0.10	-2.62	<0.01**
RC Type (Subject)	0.21	0.13	1.67	0.09
Relativizer (CL)	0.25	0.13	1.99	<0.05*
Time Region (1^st^ Cluster)	-1.56	0.02	-71.22	<0.001***
Group (YTD vs DLD)	-0.08	0.21	-0.38	0.71
Group (DLD vs AM-TD)	0.23	0.20	1.14	0.25
RC Type (Subject): Relativizer (CL)	0.21	0.25	0.81	0.42
RC Type (Subject): Time Region (1^st^ Cluster)	-0.03	0.04	-0.66	0.51
Relativizer (CL): Time Region (1^st^ Cluster)	-0.37	0.04	-8.66	<0.001***
RC Type (Subject): Group (YTD vs DLD)	-0.03	0.20	-0.13	0.90
RC Type (Subject): Group (DLD vs AM-TD)	0.14	0.20	0.70	0.48
Relativizer (CL) : Group (YTD vs DLD)	-0.16	0.20	-0.79	0.43
Relativizer (CL) : Group (DLD vs AM-TD)	0.01	0.20	0.04	0.97
Time Region (1^st^ Cluster): Group (YTD vs DLD)	-0.05	0.06	-0.90	0.37
Time Region (1^st^ Cluster): Group (DLD vs AM-TD)	-0.37	0.05	-7.19	<0.001***
RC Type (Subject): Relativizer (CL): Time Region (1^st^ Cluster)	-0.13	0.09	-1.52	0.13
RC Type (Subject): Relativizer (CL): Group (YTD vs DLD)	-0.85	0.39	-2.16	<0.05*
RC Type (Subject): Relativizer (CL): Group (DLD vs AM-TD)	0.50	0.39	1.28	0.20
RC Type (Subject): Time Region (1^st^ Cluster): Group (YTD vs DLD)	0.33	0.11	3.00	<0.01**
RC Type (Subject): Time Region (1^st^ Cluster): Group (DLD vs AM-TD)	-0.14	0.10	-1.41	0.16
Relativizer (CL): Time Region (1^st^Cluster): Group (YTD vs DLD)	0.22	0.11	1.96	0.0504
Relativizer (CL): Time Region (1^st^Cluster): Group (DLD vs AM-TD)	0.40	0.10	3.93	<0.001***
RC Type (Subject): Relativizer (CL): Time Region (1^st^Cluster): Group (YTD vs DLD)	-0.13	0.22	-0.61	0.54
RC Type (Subject): Relativizer (CL): Time Region (1^st^Cluster): Group (DLD vs AM-TD)	-0.24	0.20	-1.20	0.23

**Fig 9:**
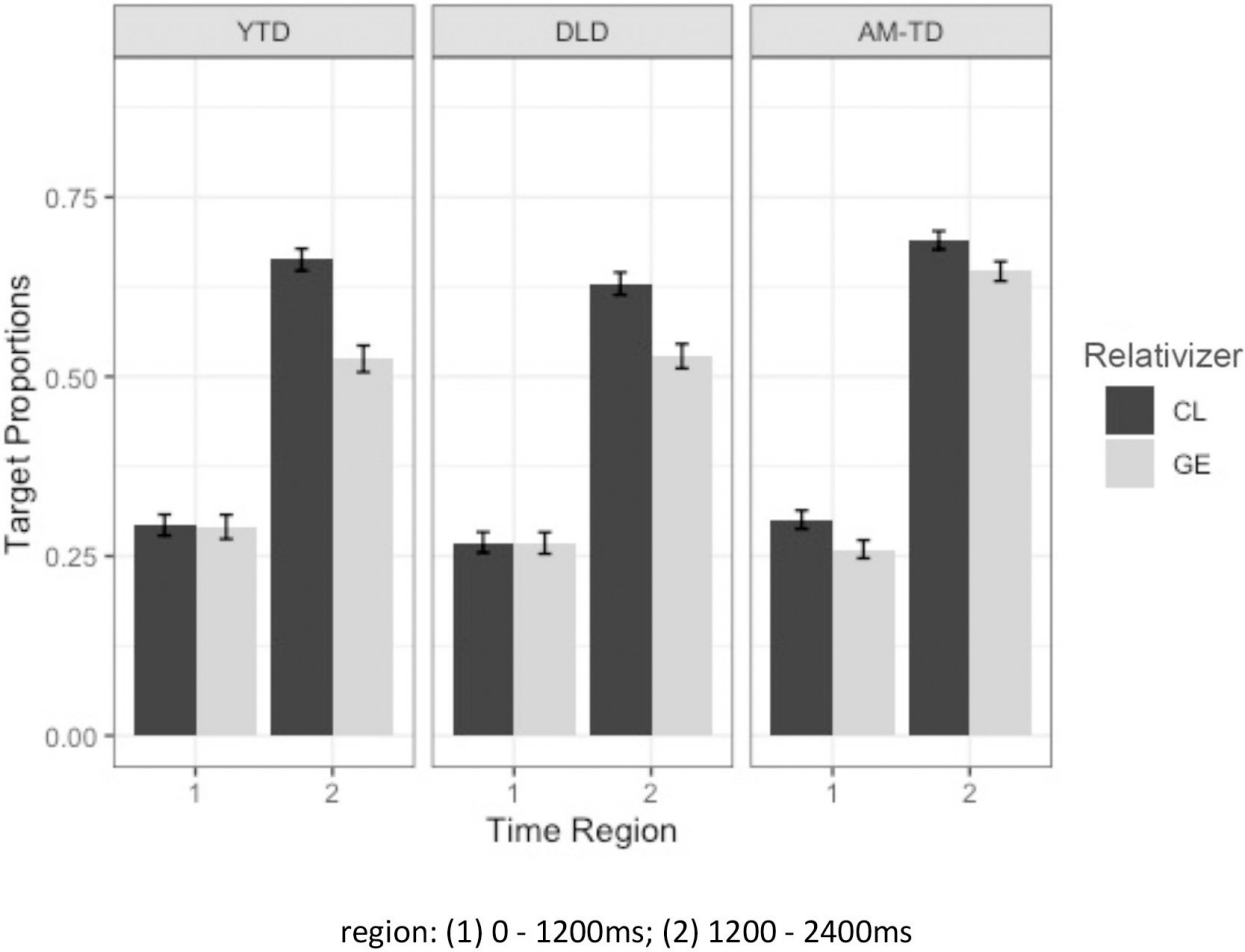
Average target proportions of looks for CL and *ge3* RCs in DLD, AM-TD, and YTD groups (error bars represent standard error).

There were two sets of significant interactions involving the DLD and AM-TD groups. Specifically, there was a two-way significant interaction between time region and group (DLD vs AM-TD), suggesting that DLD children had lower overall looks to the target than the AM-TD children across all conditions. Moreover, there was a significant three-way interaction between relativizer, time region and group (DLD vs AM-TD), indicating that the increase of target looks across time in CL vs *ge3* was not uniform between these two groups. Fig 9 plots the interaction and shows that, while the increase of target looks across time was not distinctly different between CL and *ge3* in the AM-TD children, there was a clear distinction between strategies in DLD, who showed relatively quicker convergence on the target in CL than *ge3* RCs.

**Fig 10:**
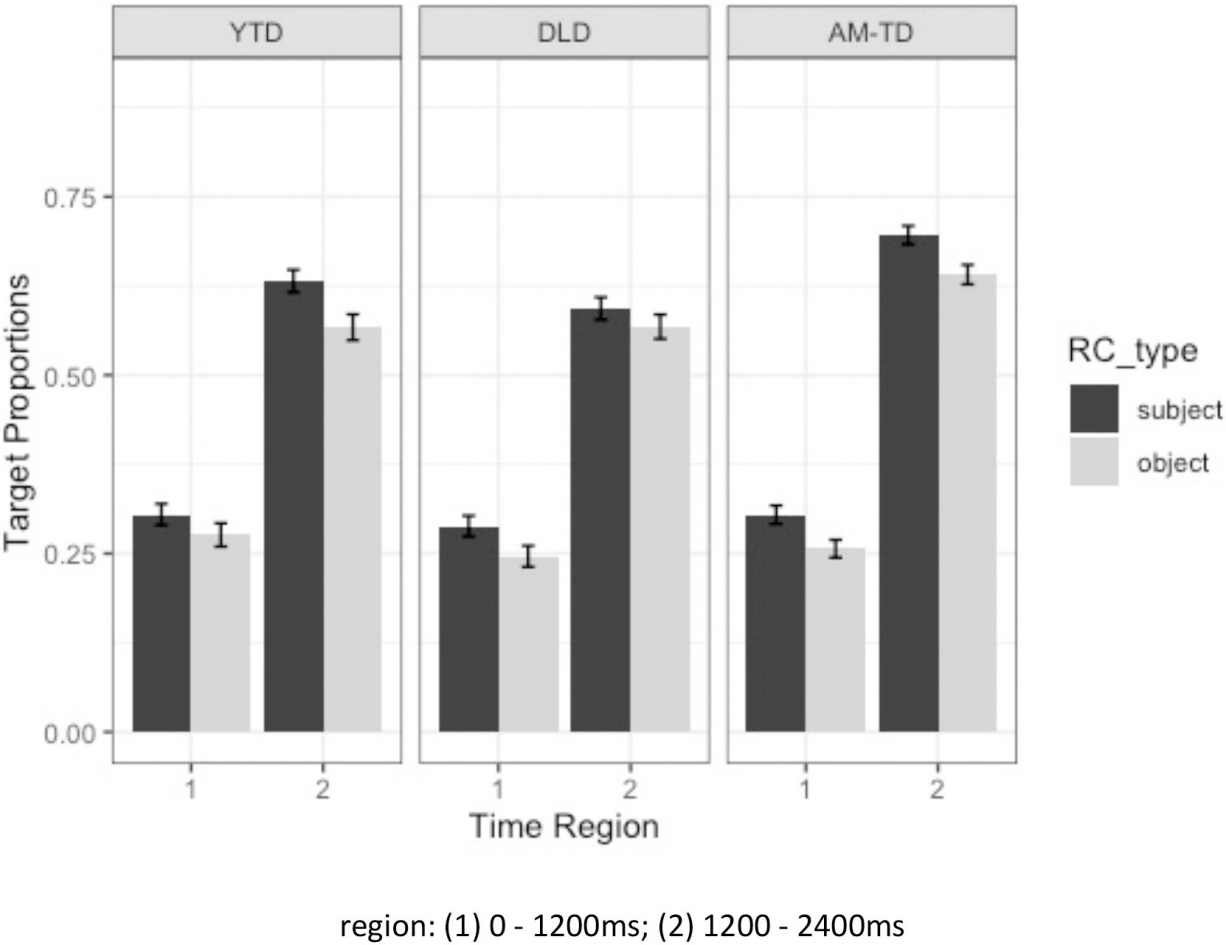
Average target proportions of looks for SRCs and ORCs in DLD, AM-TD, and YTD groups (error bars represent standard error).

Furthermore, there were two sets of significant interactions involving DLD and YTD. Specifically, there was a significant three-way interaction between RC type, time region and group (DLD vs YTD), indicating that the increase of target looks across time in SRC vs ORC was not uniform between these two groups. Fig 10 plots the interaction and shows that, while YTD showed more overall looks to the target when comprehending SRCs relative to ORCs, DLD children showed little difference between SRCs and ORCs in terms of increase of target looks over time or even a slight increase in target looks when comprehending ORCs relative to SRCs, a pattern that is not suggestive of an ORC disadvantage.

In addition, there was a significant three-way interaction between RC type, relativizer and group (DLD vs YTD), indicating that the proportions of target looks in SRC vs ORC and whether there was any distinction between CL and *ge3* was not uniform between these two groups. Fig 7 shows that the DLD children appeared to show generally higher proportions of target looks to SRCs than ORCs in the *ge3* condition but not the CL condition. This contrasted with the YTD group, who appeared to show generally higher proportions of target looks to SRCs than ORCs only in the CL condition but not the *ge3* condition.

## Discussion

In this paper we have presented the first experimental study examining RC comprehension accuracy and eye-tracking patterns during RC comprehension in Cantonese-speaking children with and without DLD. Examining Cantonese RCs in the DLD context presents a unique opportunity to test and compare domain-specific versus domain-general accounts of typical and atypical language development. Recall the three sets of predictions we tested. First, the domain-specific approach predicts a SRC over ORC in Cantonese because of the absence of structural intervention in SRCs; whereas domain-general emergentist perspective predicts a lack of SRC advantage (if not an ORC advantage) or only a weak SRC advantage (if any) from the interaction of processing factors that pull in opposite direction in Cantonese. Second, the domain-specific approach makes no explicit predictions regarding the relativizers, but domain-general approaches predict a CL over *ge3* advantage in Cantonese RC comprehension as frequency effects favor CL RCs. Third, the domain-specific representational deficit accounts [50–52] predict that DLD children will perform not only worse than their AM-TD peers, but also the language-matched YTD group in RC competence. In contrast, domain-general approaches that explain DLD as an emergent property of cognitive systems that support language acquisition [53, 54] predict that DLD children will perform worse than AM-TD but resemble the language-matched YTD group. We discuss our findings in light of these diverging predictions.

### SRCs vs ORCs

There was a uniform SRC advantage in comprehension accuracy but not in the eye-tracking data. This is a curious finding, in the sense that when children correctly interpreted the test structures there did not seem to be additional computational difficulty associated with ORCs compared to SRCs. This suggests that the SRC advantage found in the accuracy data may be explained by variables other than syntactic complexity. Our error analyses suggest that, as expected, this variable was instead the overlap between ORCs and simple transitive sentence in Cantonese. We found frequent head noun assignment errors in ORCs, where children chose the RC-internal subject erroneously as the head noun (but not in SRCs), a phenomenon that was frequently attested in the DLD (SRC: 7.7%; ORC: 38.1%) and YTD groups (SRC: 4.5%; ORC: 37.5%). This contrasted with the AM-TD children, who were equally good at SRCs (5%) and ORCs (4.5%). This error pattern has also been reported in other RC comprehension studies on Chinese-speaking children (e.g. [83–85]). Given this background, the suggestion is that the SRC advantage in the current accuracy data arose due to comprehension of ORCs being affected by the competing SVO interpretation of the test sentence. That is, the children were garden-pathed in this condition, which occurred most often in the DLD and the younger YTD children.

However, when children were not garden-pathed (i.e., when they correctly interpreted ORCs), our eye-tracking analyses indicated no significant main effect of RC type and no interaction between time region and RC type. Considering both findings from comprehension accuracy and looking behaviours, this lack of a robust subject advantage (or object disadvantage) cannot be readily explained by the domain-specific approach, which predicts a subject over object advantage for Cantonese RC processing and acquisition. Rather, these findings are more consistent with domain-general accounts, which predict that the effect of general subject prominence could be weakened by the opposing effects of shorter linear distance of filler-gap dependency, high SVO structural frequencies in children’s experience, and support from simpler known constructions (SVO transitive constructions) that are associated with ORCs in Cantonese.

Moreover, when we specifically consider the DLD children, the findings collectively do not indicate that they had a deficit specific to ORCs relative to the other two TD groups. First, recall that the significant main effect of RC type indicating SRC over ORC advantage in the accuracy measures was not specific to any group. Second, the significant interaction between RC type, time region and group (YTD vs DLD) in the eye-tracking data results revealed a lack of ORC disadvantage in Cantonese DLD when children were not garden-pathed: unlike their YTD peers who showed more overall looks to the target toy when comprehending SRCs relative to ORCs, DLD children showed little difference between the two RC types and even a slight increase in target looks when comprehending ORCs (see Fig 10). This pattern is unlike results from English and other European languages (e.g. [3] in English; [4] in Danish; [5] in Greek; see also [6] in Hebrew, a Semitic language), where the DLD children find ORCs particularly challenging relative to the TDs. Taking results from comprehension accuracy and eye-tracking data together, our study presents novel findings from a typologically distinct language, Cantonese, that do not identify ORCs causing greater difficulty for DLD children when compared to their age-matched and language-matched peers.

The lack of a robust ORC disadvantage in DLD children is similarly reported in Japanese- and Korean-speaking children with DLD [86, 87]. These typological parallels observed could be attributed to the effects of competing processing factors in these languages: in Japanese and Korean, ORCs, rather than SRCs, resemble simple transitive SOV constructions in terms of having subject before object in their word order configuration The typological parallels of a lack of a robust ORC disadvantage in Cantonese, Japanese and Korean-speaking children with DLD are attributed to a common effect arising from ORCs sharing similarity with the canonical transitive constructions in these languages, that pull in opposite direction from general subject prominence: Cantonese ORCs resemble the simple SVO transitives, whereas Japanese and Korean ORCs resemble the simple SOV transitives in terms of having subject before object in the word order configuration (c.f. [86]). However, unlike Cantonese ORCs, Japanese and Korean ORCs do not receive further support from the linear distance factor which would predict no processing difference between SRCs and ORCs in these two languages (c.f. [19]). As such, support from simpler known constructions that favor ORCs pull in opposite direction from general subject prominence. The scenario is very different in languages like English, where factors such as subject prominence, linear distance, support from simpler known constructions, and structural frequencies in the input would all coalesce to create a strong bias favouring SRCs over ORCs. Moreover, the contrast between the lack of robust ORC disadvantage in Cantonese, Korean and Japanese DLD and the robust SRC advantage in English and other European languages concurs with Leonard & Kueser [88]’s cross-linguistic observation that DLD children find syntactic structures easier to acquire if they resemble simpler known constructions with the same canonical word order.

### CL vs ge3

There was a CL over *ge3* advantage in comprehension accuracy and children’s looking preference during RC comprehension. In the eye-tracking analyses there was a significant interaction between relativizer and time region, showing that all children had more target looks in the CL RCs condition than the *ge3* RCs condition, upon hearing the head noun (i.e. at the second phase). This suggests that children converged to the target looks in the CL RCs condition faster and with more certainty than the *ge3* RCs condition. This pattern of results is consistent with the domain-general suggestion that frequently occurring structural patterns should be processed more easily because they are earlier acquired. In this instance, children’s greater familiarity with CL RCs appears to have eased their processing of the target sentences. The result is inconsistent with the domain-specific approach, whose focus on syntactic derivation at the exclusion of frequency, does not predict a difference.

The eye-tracking data revealed some differences in the children’s processing of CL and *ge3* RCs. There was a significant three-way interaction between relativizer, time region and group (DLD vs. AM-TD), indicating that the AM-TD children processed both relativizers similarly, whereas the DLD children showed a clear advantage for CL over *ge3* RCs. This specific pattern of findings could also be interpreted in light of domain-general perspectives. Notably, if children with DLD have weaker processing and learning abilities that slow their development [53–56], then they will find lower frequency forms like the *ge3* RCs more difficult.

### DLD vs TD children

Consistent with the robust cross-linguistic evidence in the DLD literature, Cantonese DLD children performed significantly worse than their AM-TD peers, scoring lower in RC comprehension accuracy. Moreover, our eye-tracking findings revealed a significant two-way interaction between time region and group (DLD vs AM-TD), indicating that the increase in target looks over time was smaller in DLD relative to AM-TD in general. This result therefore suggests that these DLD children displayed a slower processing speed than age-matched TD in general, a phenomenon that has also been well-documented in the literature [89–91]. The finding of slower processing speed is again compatible with how the domain-general limited capacity processing accounts of DLD [53] conceptualize the nature of difficulty in DLD: children with DLD having limited processing capacity could find it more challenging to process complex sentences like RCs that are cognitively taxing, resulting in slower processing speed during comprehension.

Importantly, our results also indicated that DLD children were not worse than YTD children in comprehension accuracy, and there is no evidence from their looking behaviours that DLD children were worse than YTD in processing RCs. This pattern of findings therefore suggests that these DLD children did not show a specific difficulty with RC comprehension relative to their language-matched YTD peers, contra the domain-specific representation deficit accounts of DLD [50–52]. Our findings are instead consistent with the domain-general perspectives predicting a global delay (rather than a specific difficulty) in DLD, where DLD children could resemble their language-matched YTD peers in RC comprehension.

Some limitations to our study merit comment. Firstly, our study had relatively small samples across three participant groups. While these sample sizes are common in studies of DLD that use matched designs, it is likely that our study did not have enough power to observe all differences across conditions and groups, and so we acknowledge that they await replication. Additionally, we analysed our looking time behaviour in a fairly simple manner. This was intentional: given the number of groups and conditions, analysing the data at a finer grain had the potential to result in too many false positive and negatives. Therefore, future studies should aim to test bigger sample sizes to facilitate the analysis of moment-by-moment parsing decisions.

To conclude, our study is the first to examine RC comprehension and eye-tracking looking preference during RC comprehension in the Chinese DLD literature. Empirically, our findings demonstrated that RC comprehension is indeed vulnerable in Cantonese-speaking DLD children relative to their age-matched TD peers. Theoretically, we examined three dimensions, namely SRCs vs ORCs, CL vs *ge3* RCs, and DLD vs TD children, where domain-specific and domain-general theories make diverging predictions. The current findings pose challenges to the domain-specific structural approaches specified in structural intervention [9] in combination with representational deficit account of DLD [50–52], and are better explained by domain-general perspectives of both typical [19, 44, 49] and atypical language development (i.e. Montgomery and Evan [53]’s domain-general limited capacity processing account of DLD).

## Supporting information

S1 Appendix Cantonese RC stimuli—referent selection task. (DOCX)
