# Supplementary material for: Relative clause comprehension in Cantonese-speaking children with and without developmental language disorder
Source: PLoS One. 2023 Nov 7;18(11):e0288021. doi: 10.1371/journal.pone.0288021 (PMC10629646; doi:10.1371/journal.pone.0288021)
Supplement: S1 Appendix — (DOCX) [file pone.0288021.s001.docx]

# **S1 Appendix. Cantonese RC stimuli - Referent selection task.**

**Can you pick up [relative clause] head noun?**

**Subject CL RCs**

1. 追 獅子 嗰 隻 狗仔

zeoi1 si1zi2 go2 zek3 gau2zai2

chase lion that CL dog

‘the dog that chased the lion’

2. 踢 斑馬 嗰 隻 熊人

tek3 baan1maa5 go2 zek3 hung4jan2

kick zebra that CL bear

‘the bear that kicked the zebra’

3. 抹 豬仔 嗰 隻 馬騮

maat3 zyu1zai2 go2 zek3 maa5lau1

wipe pig that CL monkey

‘the monkey that wiped the pig’

4. zit1 馬騮 嗰 隻 牛牛

zit1 maa5lau1 go2 zek3 ngau4ngau2

tickle monkey that CL cow

‘the cow that tickled the monkey’

**Object CL RCs**

1. 馬仔 推 嗰 隻 狗仔

maa5zai2 teoi1 go2 zek3 gau2zai2

horse push that CL dog

‘the dog that the horse pushed’

2. 老虎 咬 嗰 隻 熊人

lau5fu2 ngaau5 go2 zek3 hung4jan2

tiger bite that CL bear

‘the bear that the tiger bit’

3. 羊仔 摸 嗰 隻 馬騮

joeng4zai2 mo2 go2 zek3 maa5lau1

sheep touch that CL monkey

‘the monkey that the sheep touched’

4. 老虎 餵 嗰 隻 牛牛

lau5fu2 wai3 go2 zek3 ngau4ngau2

tiger feed that CL cow

‘the cow that the tiger fed’

**Subject *ge3* RCs**

1. 舐 斑馬 嘅 獅子

lam2 baan1maa5 ge3 si1zi2

lick zebra ge3 lion

‘the lion that licked the zebra’

2. 撞 熊人 嘅 老虎

zong6 hung4jan2 ge3 lou5fu2

bump bear ge3 tiger

‘the tiger that bumped the bear’

3. 咬 牛牛 嘅 大象

ngauu5 ngau4ngau2 ge3 daai6zeong6

bite cow ge3 elephant

‘the elephant that bit the cow’

4. 推 長頸鹿 嘅 老虎

teoi1 ceong4geng2luk5 ge3 lou5fu2

push giraffe ge3 tiger

‘the tiger that pushed the giraffe’

**Object *ge3* RCs**

1. 熊貓 舐 嘅 獅子

hung4maau1 lam2 ge3 si1zi2

panda lick ge3 lion

‘the lion that the panda licked’

2. 大象 追 嘅 老虎

daai6zoeng6 zeoi1 ge3 lou5fu2

elephant chase ge3 tiger

‘the tiger that the elephant chased’

3. 豬仔 踢 嘅 牛仔

zyu1zai2 tek3 ge3 ngau4zai2

pig kick ge3 cow

‘the cow that the pig kicked’

4. 大象 撞 嘅 長頸鹿

daai6zoeng6 zong6 ge3 coeng4geng2luk5

elephant bump ge3 giraffe

‘the giraffe that the elephant bumped’
